# Antimicrobial Efficacy of Single-Walled Carbon Nanotubes and Their Combinations Against Enterococcus faecalis Assessed Using the Agar Diffusion Method: An In Vitro Study

**DOI:** 10.7759/cureus.74626

**Published:** 2024-11-27

**Authors:** Binita Saha, Kingston Chellapandian, Vijay Venkatesh

**Affiliations:** 1 Department of Conservative Dentistry and Endodontics, SRM Kattankulathur Dental College and Hospital, Chennai, IND; 2 Department of Conservative Dentistry and Endodontics, SRM Kattankulathur Dental College, Chennai, IND

**Keywords:** anti-microbial properties, calcium hydroxide, chlorhexidine, enterococcus faecali, intracanal medicaments, irrigants, multi-walled carbon nanotubes, nanoparticle, single-walled carbon nanotubes

## Abstract

Introduction: This study aimed to evaluate the antimicrobial efficacy of single-walled carbon nanotubes when combined with the commonly used intracanal medicaments by checking their zone of inhibition against *Enterococcus faecalis*.

Materials and methods: The test materials were divided into five different groups, namely, Group I: single-walled carbon nanotubes; Group II: calcium hydroxide; Group III: chlorhexidine; Group IV: single-walled carbon nanotubes + calcium hydroxide; and Group V: single-walled carbon nanotubes + chlorhexidine. Five sterile Petri plates per group were inoculated with *Enterococcus faecalis *(*E. faecalis*); wells were made in the plates, one on each side, and a volume of 50 microliters of each solution was dispensed into individual wells using a pipette. The specimens were placed in an incubator at 37°C for a duration of 48 hours. The area of inhibition surrounding each well was documented and assessed. The statistical analysis was conducted using the Kruskal-Wallis test and the Kolmogorov-Smirnov test.

Results: Single-walled carbon nanotubes combined with chlorhexidine have shown the highest antimicrobial efficacy against *Enterococcus faecalis* in comparison to all the test groups.

Conclusion: This study showcases the antimicrobial effectiveness of a blend of single-walled carbon nanotubes and chlorhexidine solution. It may be developed as a potent intracanal medicament in the future.

## Introduction

Carbon is the fourth most abundant chemical element present in the universe by mass. Various studies have shown that carbon nanotubes, fullerenes, etc. have effective antimicrobial properties. As per the literature, the bonding of bacteria with the carbon nanoparticle results in the immediate interaction between the cells and carbon nanomaterials, ultimately resulting in the death of the cell [1]. It has been uncovered that the antibacterial activity of carbon nanomaterials is influenced by their size and surface area. Specifically, enhancing the activity in interacting with bacteria is achieved by increasing the surface area through the reduction of nanoparticle size. Carbon nanotubes damage the cell walls and membranes of microorganisms. Additionally, they can induce oxidative stress within the cell, which can result in the cell's biological death [2]. Carbon nanotubes are carbon structures with a nano-sized, hollow cylindrical shape, initially synthesized by Lijima in 1991 [3]. The initial evidence of potent antimicrobial effects on *Escherichia coli* (*E. coli*) by single-walled carbon nanotubes was presented by Kang et al. in 2007 [3,4].

Studies have shown that individual, single-walled structures of carbon nanotubes have far superior properties than multi-walled structures [3, 4]. The integrity of the cell membrane, metabolic activities, and cytology of *E. coli* were impacted by first-hand association with carbon nanotubes. The authors suggest that the penetration of single-walled carbon nanotubes into the cell wall is more effective compared to multi-walled carbon nanotubes due to their reduced size and diameter. Additionally, the enhanced cell-surface interaction was facilitated by the increased surface area of the single-walled carbon nanotubes [1,4].

Mechanical debridement, along with cleaning, are crucial factors that determine the favorable result of a root canal treatment. However, the intricate structure of the root canal allows certain bacteria to harbor in many areas in the canal. *Enterococcus faecalis* (*E. faecalis*), a type of anaerobic cocci with a positive gram stain, is a primary contributor to failures in endodontic treatments. A prior investigation revealed that it was present in 18% of initial infections that occur in the canal and a significant 67% of cases where endodontic procedures were unsuccessful [5]. *Enterococcus faecalis* initiates the immune response of the body and hampers the lymphocyte role. It demonstrates resilience in suboptimal nutritional conditions, surviving as a singular microorganism. Additionally, it can infiltrate into the tubular structure of dentin and create biofilms. The formation of a polymeric matrix outside the cell by *E. faecalis* makes bacterial biofilms highly resistant to standard irrigants [5]. To maintain a bacteria-free environment, intracanal medicaments are employed against root canal bacteria, proving effective against microbes that persist despite mechanical instrumentation and cleaning.

Studies have shown that the growth of biofilm was reduced by 81.19% after 48 hours of incubation when exposed to 50 μg/ml, one of the single-walled carbon nanotubes, and the production of biofilm was completely inhibited at concentrations of 200 μg ml, one or higher. Single-walled carbon nanotubes exhibited greater effectiveness in inhibiting biofilm formation when present in the early phases of biofilm production [6].

Calcium hydroxide and chlorhexidine are the two most commonly used intracanal medicaments used in endodontic practice. Calcium hydroxide is the most frequently used intracanal medicament because its high alkaline pH appears to inhibit bacterial growth [7]. Several comparative studies using various concentrations of chlorhexidine have shown that a 2% solution has the highest substantivity against root canal bacteria. However, in recent years, bacterial resistance to traditional medicaments has led to the failure of root canal-treated teeth [8].

Carbon nanotubes, with their unique structure, exhibit a range of fascinating properties that span mechanical, physical, chemical, and biological dimensions. Carbon nanotubes are renowned for their remarkable mechanical attributes. Their tensile strength is notably enhanced due to a senary arrangement, a testament to their robust structure. They exhibit a malleability akin to rubber, allowing them to bend and flex without losing their integrity. Their ductility is also impressive, falling within a range of 8% to 12%, which further underscores their flexibility [9]. Overall, their exceptional mechanical strength makes them incredibly resilient under stress. On the physical front, carbon nanotubes are characterized by their extensive surface area, which is a key feature in many of their applications. They are incredibly light, contributing to their utility in various lightweight applications. Their high thermal stability ensures that they maintain performance even at elevated temperatures, while their reduced density makes them an efficient material choice for a range of uses. Chemically, carbon nanotubes stand out for their efficient heat conduction, which is a result of their unique atomic arrangement. The superior bonding among the carbon atoms, organized in hexagonal rings, contributes to their overall stability and strength. This arrangement allows them to conduct heat effectively and maintain structural integrity. In the biological realm, carbon nanotubes exhibit properties that can be both beneficial and challenging [9]. They are known for their improved antimicrobial characteristics, which can be advantageous in medical applications. They possess the ability to perforate bacterial cell membranes, making them a potential tool in combating infections. However, they can also trigger inflammatory and fibrotic responses under certain conditions, highlighting the need for careful consideration in their application [9].

Overall, the diverse properties of carbon nanotubes make them a subject of significant interest across various fields, from materials science to medicine [9]. The objective of this study is to integrate single-walled carbon nanotubes with conventional medicaments. in order to improve their properties and check their effectiveness against *E. faecalis*.

## Materials and methods

The research was planned to be an in vitro assessment to ascertain the antimicrobial effectiveness of two root canal medicaments alone and when used in combination with single-walled carbon nanotubes against *E. faecalis*. The nanoparticles were obtained from the nanotechnology lab situated in Jamshedpur, India. Culture plates, agar medium, and other pieces of equipment for bacteriological evaluation were obtained from Hi-Media Laboratories, Mumbai, India. The bacterial strain to be subcultured was obtained from the central lab of microbiology at SRM Kattankulathur Dental College and Hospital, Chennai, India.

Test materials

The test materials were categorized under five groups: Group I consisted of single-walled carbon nanotubes (Techinstro, Nagpur, India) solution; Group II contained solutions of calcium hydroxide (Prime Dental Products, Thane, India); Group III contained chlorhexidine-2% chlorhexidine (Prevest Dentpro, Jammu, India), respectively. Group IV contained combined solutions of single-walled carbon nanotubes and calcium hydroxide; Group V contained single-walled carbon nanotubes combined with chlorohexidine (2%).

Test organism

The test organism in this study was *Enterococcus faecalis* (ATCC 29212).

Preparation of test solutions

For Group I, single-walled carbon nanotubes with a diameter of 1.8 nanometers, a length of 5 micrometers, 0.1 g/cm³ of bulk density, and a surface area of 490 m²/g were used; 2-3 mg of (powder) nanoparticles were mixed with 4 ml of solvent made of chloroform, benzene, and acetone (1:2:1) (molecular biology grade). For Group II, 100 mg/ml of calcium hydroxide was mixed with 4 ml of distilled water (molecular biology grade). For Group III, 3 ml of 2% chlorhexidine was mixed with 2 ml of distilled water (molecular biology grade). Group IV solutions were prepared by combining 2.5 ml of the calcium hydroxide stock with 2.5 ml of carbon nanotube stock. Group V contained 2.5 ml of the chlorhexidine stock, which was mixed with 2.5 ml of nanotube stock.

Characterization of nanoparticles

**Figure 1:**
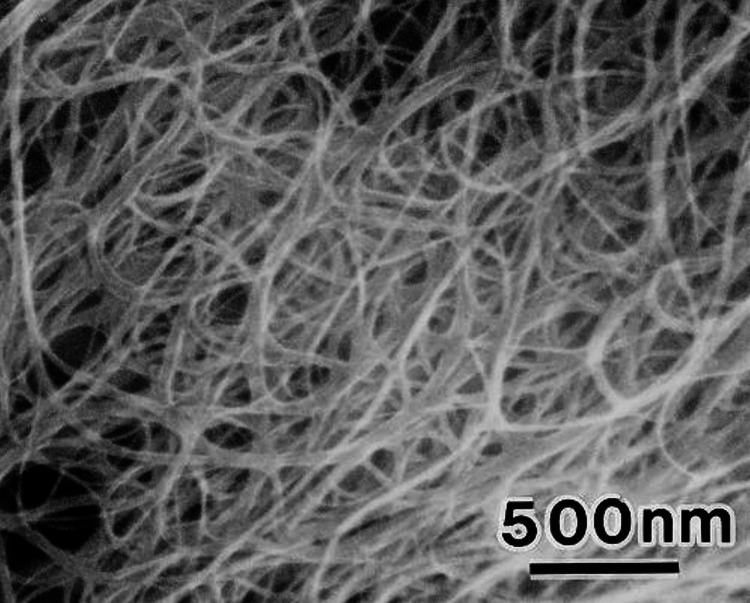
Scanning electron microscope analysis of single-walled carbon nanotubes + calcium hydroxide

**Figure 2:**
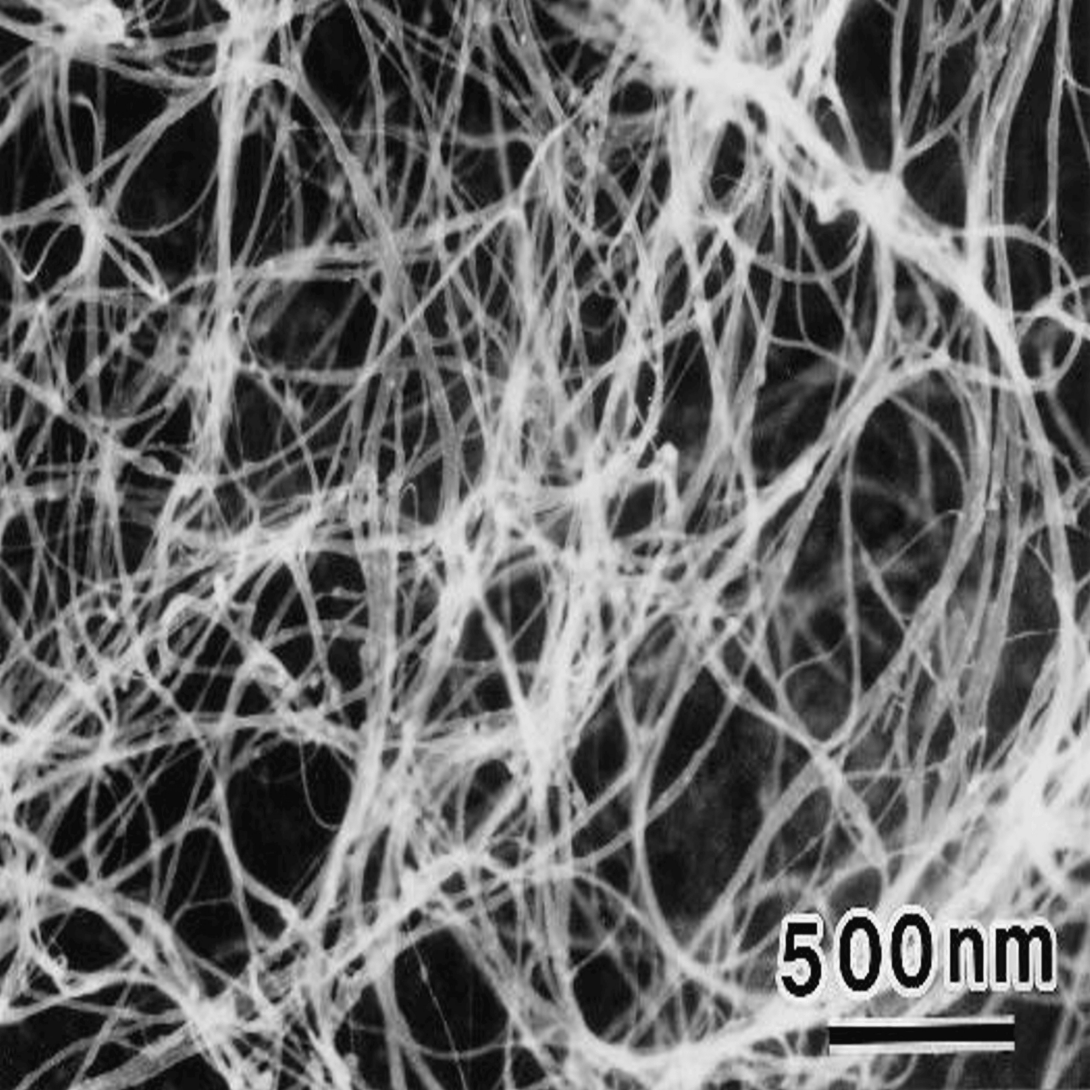
Scanning electron microscope analysis of single-walled carbon nanotubes + chlorhexidine

Scanning electron microscopy (SEM) revealed the presence of long cylindrical carbon nanotubes along with small, scattered particles of calcium hydroxide and chlorhexidine (Figures 1-2). 

**Figure 3:**
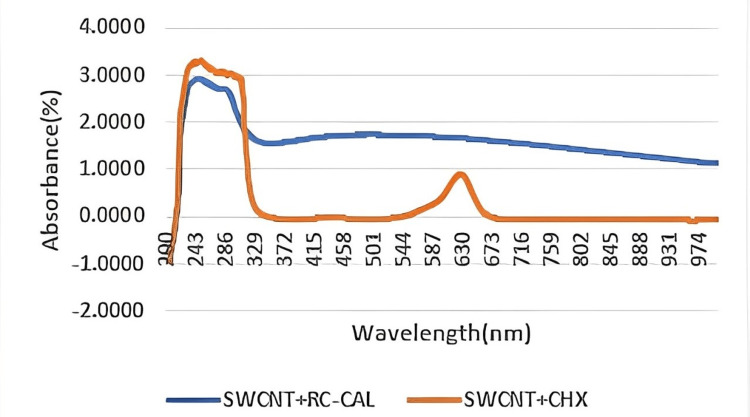
Ultraviolet spectroscopy of single-walled carbon nanotubes (SWCNT) + calcium hydroxide (RC-CAL) and single-walled carbon nanotubes + chlorhexidine (CHX)

Ultraviolet-spectroscopy has strong absorbance at a peak of 246 for single-walled carbon nanotubes + chlorhexidine and a peak of 228 for single-walled carbon nanotubes + calcium hydroxide. Figure 3 demonstrates the graph generated by the UV-spectroscopy results of both solutions.

Preparation of microorganisms

The microorganisms for the experiment were introduced into a tube containing sterile peptone water and incubated for 30 minutes. Mueller-Hinton agar medium was used for the bacterial culture. The microorganisms were streaked into the Petri dishes by the lawn culture method. A total of five samples for each solution were prepared and categorized into groups.

Assessing the antibacterial activity by agar well diffusion method

After drying the streaked Petri plates, two wells, each with a diameter of 6 mm, were created on every plate. The plates were labeled and distributed in a way that each plate consisted of two solutions, one on each side, to prevent any misinterpretation or overlap due to crowding. Fifty microliters of each solution were dispensed into individual wells using a pipette in the uniformly distributed plates. The Petri dishes were placed in an incubator at a temperature of 37°C for a duration of 48 hours. The extent of inhibition was gauged using a scale and documented.

Statistical analysis

The data analysis process was meticulously conducted, utilizing Microsoft Office Excel 2021 (Microsoft Corp., Redmond, WA) to tabulate the values and IBM SPSS Statistics software version 23 (IBM Corp., Armonk, NY) for comprehensive statistical analysis. The initial step involved a descriptive analysis, which provided an in-depth exploration of the nature of the collected data. This approach facilitated the identification of key trends, central tendencies, and variability within the dataset. 

The normality of the data distribution was assessed using the Kolmogorov-Smirnov test. It is useful for ascertaining whether the data adheres to a normal distribution, a critical assumption in numerous analyses. The results of this test provided insights into the conformity of the data to the normal distribution, enabling us to make informed decisions about the appropriateness of subsequent statistical procedures.

Furthermore, to compare the different groups within the dataset, the Kruskal-Wallis test was implemented. By applying this test, we managed to perceive any significant variations amid the groups, allowing for a robust evaluation of the data across various conditions or variables.

## Results

In the purview of the present study, Table 1 shows the intergroup comparisons of the five test solutions used against the test organism *E. faecalis*. Group V (single-walled carbon nanotubes + chlorhexidine) showed the largest zone diameter, followed by Group III (chlorhexidine) and Group I (single-walled carbon nanotubes). There were no zones visible in Group II (calcium hydroxide); Group IV (single-walled carbon nanotubes + calcium hydroxide) showed small scattered areas of inhibition, but no proper zones were visible.

**Table 1:** Intergroup comparison of test solutions and a comprehensive overview of mean rank

Group	N	Mean rank
Group I: Single-walled carbon nanotubes	5	13.00
Group II: Calcium hydroxide	5	5.50
Group III: Chlorohexidine (2%)	5	18.00
Group IV: Single-walled carbon nanotubes + calcium hydroxide	5	5.50
Group V: Single-walled carbon nanotubes + chlorohexidine	5	23.00

Table 1 also provides a comprehensive overview of the mean rank of the zone of inhibition for each solution, serving as a valuable tool for comparing the efficacy of different solutions. The mean rank scores offer a quantitative measure that can be employed to assess the relative impact of each solution on inhibiting the tested variables.

To ascertain whether these solutions exhibit statistically significant differences in their zone of inhibition, the test statistics table becomes instrumental. Table 2 summarizes the outcomes of the Kruskal-Wallis H test. This statistical test goes beyond a basic comparison of means and is particularly useful when dealing with non-normally distributed data or ordinal variables. It provides essential information, including the chi-squared statistic, degrees of freedom, and the statistical importance of the test.

**Table 2:** Kruskal-Wallis test results for zone of inhibition significance

Test statistics	Zone of inhibition
Chi-square	23.602
Degrees of freedom (df)	4
Asymptotic significance (Asymp. sig.)	.0001

The Kruskal-Wallis H test conducted on the data yielded a noteworthy outcome, indicating a statistically notable variance in the inhibition zone among the different solutions (χ2(4) = 23.602, p = 0.0001).

The mean rank scores corresponding to each group further elucidate the variations observed. Notably, Group I exhibited a mean rank score of 13, Groups II and IV had a mean rank of 5.50, Group III recorded a mean rank of 18.00, and Group V secured a mean rank of 23. These scores provide a nuanced understanding of the relative performance of each solution, contributing to the overall interpretation of the Kruskal-Wallis test results.

In summary, the integration of mean rank scores, the Kruskal-Wallis H test, and the subsequent analysis of test statistics (as presented in Table 2) collectively affirm a statistically noteworthy variance in the inhibition zone among the examined solutions. This empirical evidence serves as a valuable foundation for further discussions and conclusions regarding the comparative effectiveness of the different solutions in inhibiting the observed variables.

## Discussion

The enduring success of root canal treatment relies heavily on the thorough removal of the intracanal bacteria [5]. The application of intracanal medicament is required for eradicating microbial flora during the root canal treatment process. Failure to achieve this elimination may turn into a chronic periapical infection and the need for subsequent retreatment [10,11]. Research indicates that *E. faecalis* is commonly the primary and tenacious organism responsible for stubborn endodontic infections [10,12]. The intracanal medicament is expected to interrupt biofilms and suppress any remaining bacteria till the duration it is placed inside. It should also enter the tubular structure of dentin without inducing any harm to the peri-radicular structures [13]. Notably, *E. faecalis* possesses a high tolerance to harsh environments, primarily due to its resistance to alkali and the presence of different virulence elements. These elements, common to other organisms as well, contribute to the exacerbation of the overall condition. The organism demonstrates genetic polymorphism, the capacity to endure extended spans of food deprivation, and the capacity to bind to protein structures that enable it to adhere to the dentinal surface [1,3,5,9,10,11,12,14-17].

**Figure 4:**
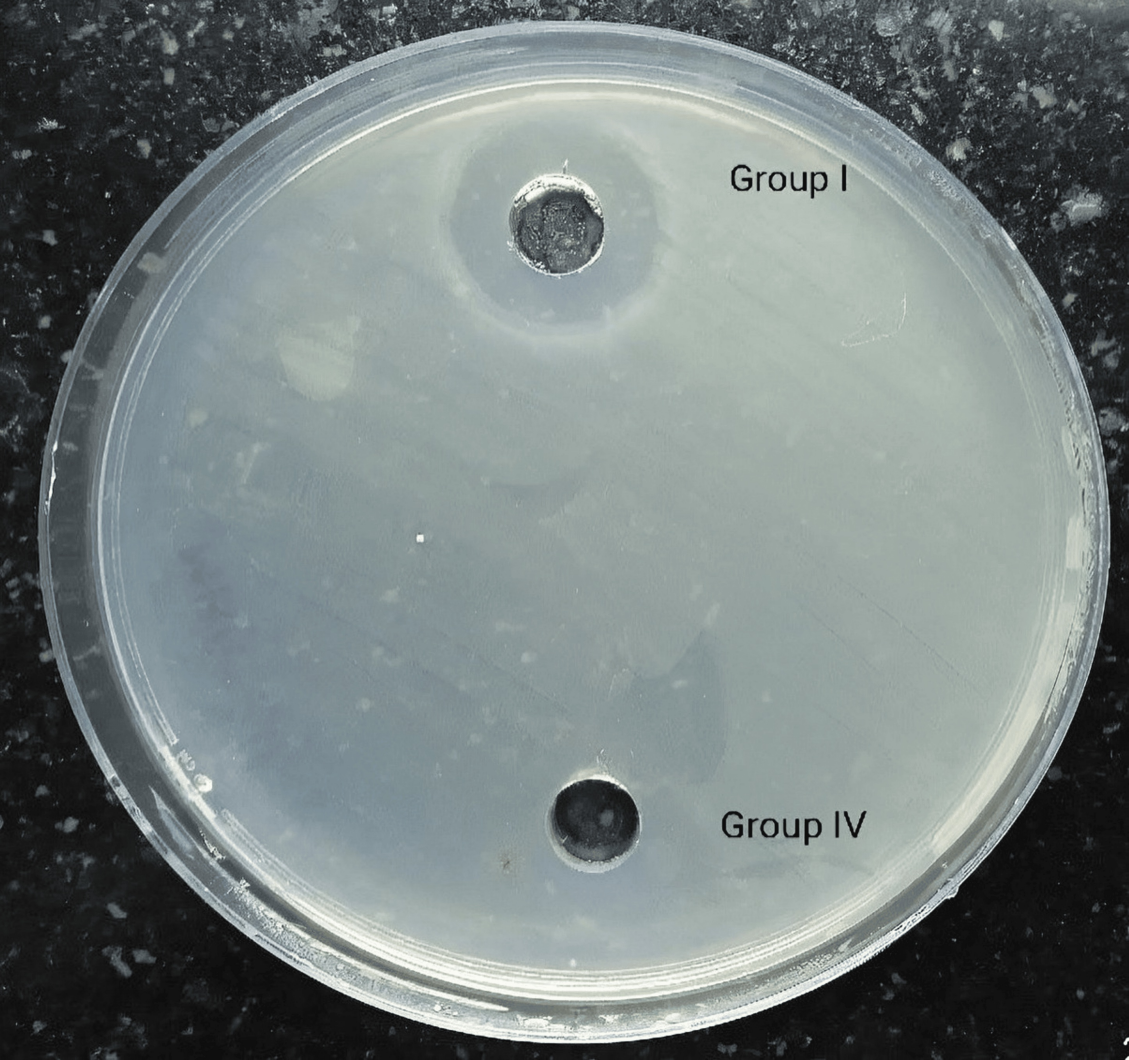
Antimicrobial activity of single-walled carbon nanotubes (Group I) and single-walled carbon nanotubes + calcium hydroxide (Group IV)

In this study, single-walled carbon nanotubes (Group I) showed a fairly good zone of inhibition, measuring 18 mm on average among all samples (Figure 4).

**Figure 5:**
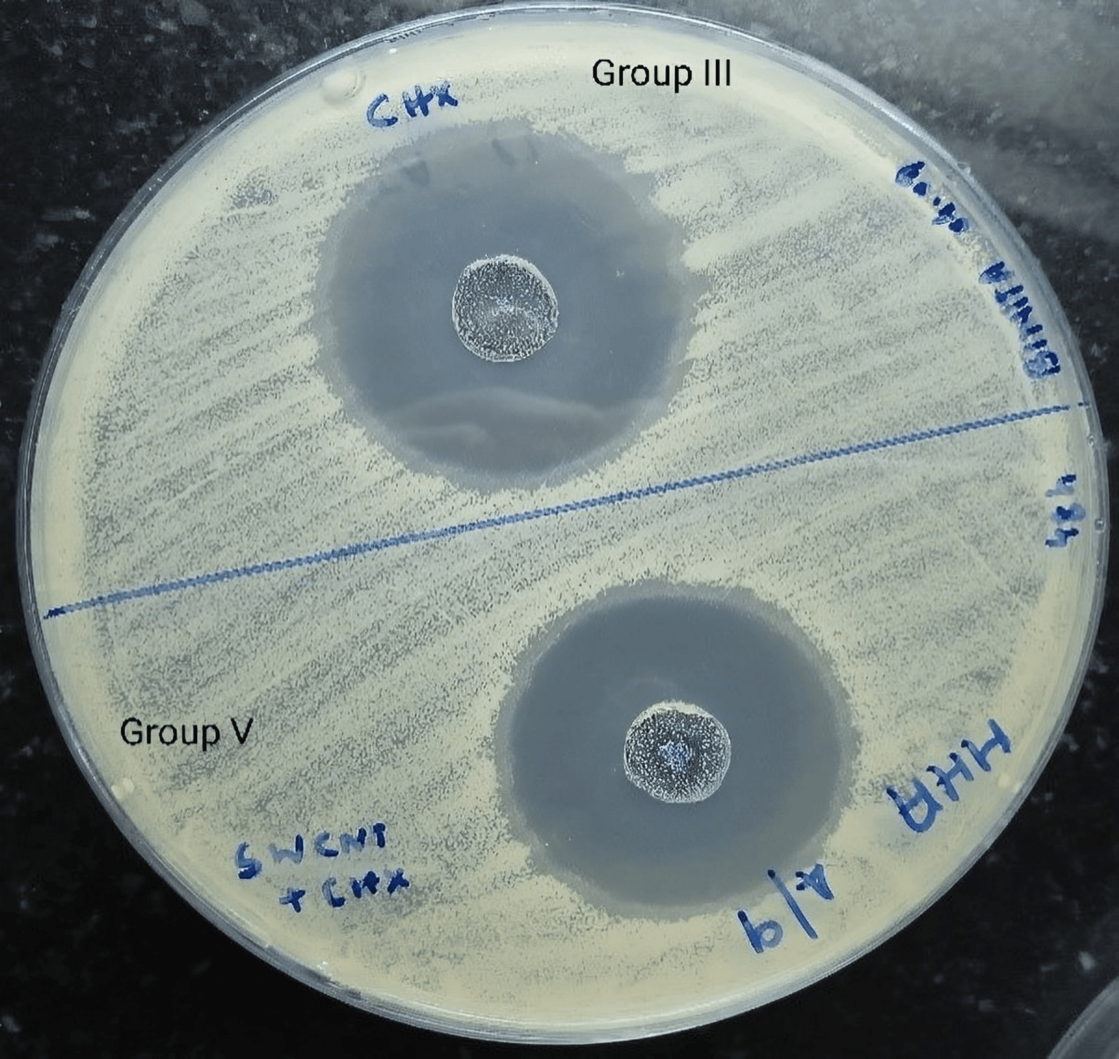
Antimicrobial activity of chlorhexidine (Group III) and single-walled carbon nanotubes + chlorhexidine (Group V)

Single-walled carbon nanotubes + calcium hydroxide (Group IV) showed no clearly demarcated zone of inhibition. Chlorhexidine (Group III) and single-walled carbon nanotubes + chlorhexidine (Group V) showed clear and well-circumscribed zones of inhibition measuring 30 mm in diameter in Group III and 32-34 mm in diameter in Group V (Figure 5).

**Figure 6:**
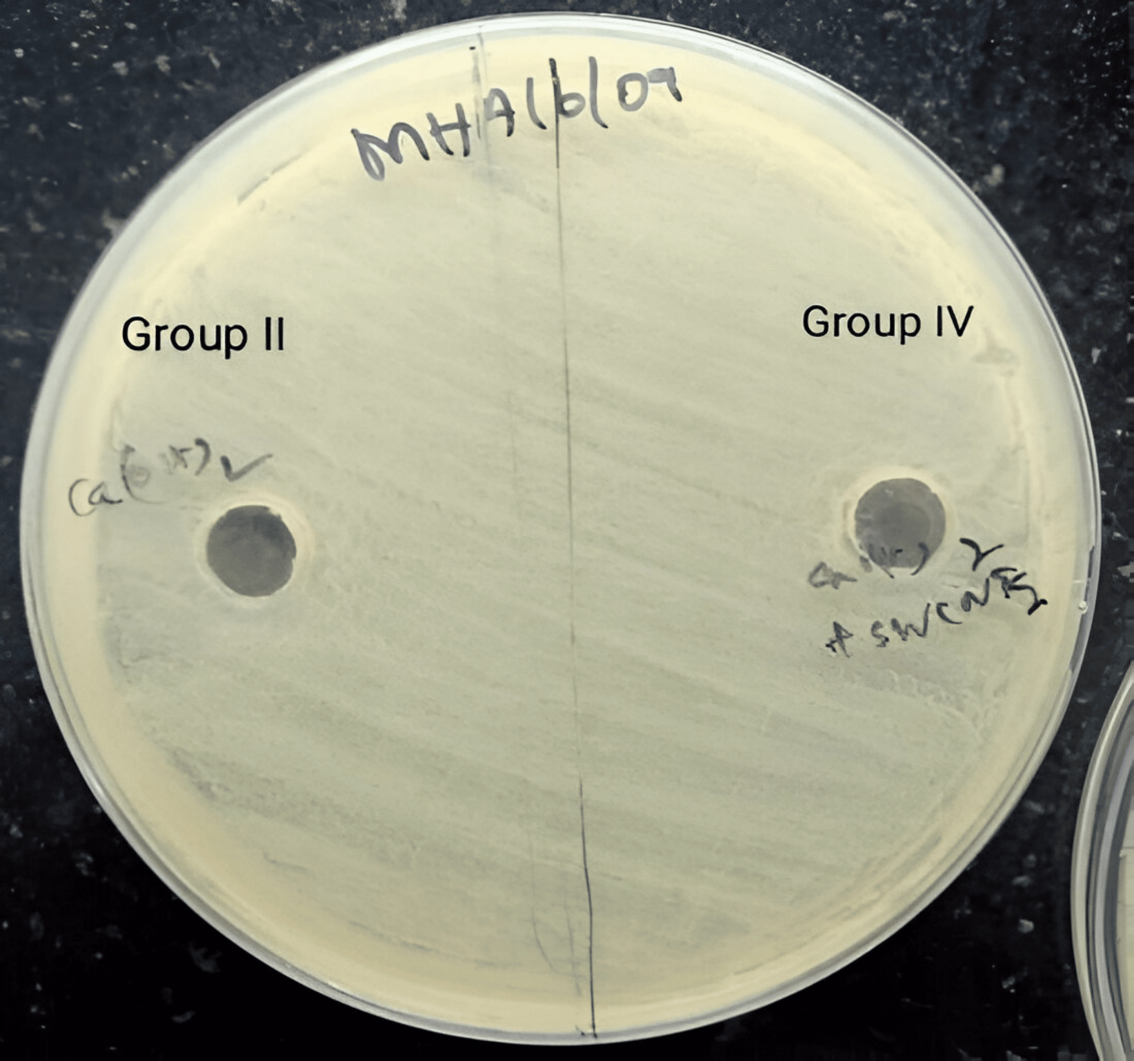
Antimicrobial activity of calcium hydroxide (Group II) and single-walled carbon nanotubes + calcium hydroxide (Group IV)

Calcium hydroxide (Group II) and single-walled carbon nanotubes + calcium hydroxide (Group IV) showed no clear zone of inhibition (Figure 6).

Single-walled carbon nanotubes initiate a somatic interaction with the cellular membrane, leading to the formation of aggregates between cells and the nanotubes. This interaction further results in the disruption of the cell membrane. Additionally, these nanotubes exhibit stability and a high potential for functionalization [17]. The intracanal medicaments frequently employed include calcium hydroxide and chlorhexidine. Some studies have raised doubts about the effectiveness of calcium hydroxide in bringing down the bacterial load due to dentin’s capacity to act as a buffer, the resilience of *E. faecalis* to hydroxyl ions, and its limited dispersibility and circulating characteristics [18-21]. In earlier research, different carriers have been incorporated into calcium hydroxide to enhance its properties. The efficacy of chlorhexidine, particularly against *E. faecalis*, is well-established; its effectiveness is linked to its biguanide nature, which engages with the surface of the bacteria that is negatively charged [13].

Several studies have examined and compared the effectiveness of chlorhexidine, calcium hydroxide as a single agent, and calcium hydroxide when mixed with other intracanal medicaments. However, there has been no evaluation of the combined action of chlorhexidine and calcium hydroxide with single-walled carbon nanotubes as an intracanal medicament.

Limitations

A further prospective in-vivo study is required to evaluate and monitor the long-term effects of the modified medicaments in the root canal.

## Conclusions

In this study, the agar well diffusion method was used to assess the antimicrobial strength. The results of the study show the area of inhibition formed against* E. faecalis* was the largest by the single-walled carbon nanotubes-chlorhexidine group (Group V). The chlorhexidine solution also created a region of inhibition against the microorganism utilized in this research. The study indicated that the antimicrobial efficacy of the single-walled carbon nanotubes-chlorhexidine solution surpassed that of the single-walled carbon nanotubes solution and the 2% chlorhexidine used independently. The single-walled carbon nanotubes-calcium hydroxide solution group has shown better antibacterial properties if compared to the calcium hydroxide group independently, but it is still negligible.
